# Utility–Privacy Trade-Off in Distributed Machine Learning Systems

**DOI:** 10.3390/e24091299

**Published:** 2022-09-14

**Authors:** Xia Zeng, Chuanchuan Yang, Bin Dai

**Affiliations:** 1 School of Information Science and Technology, Southwest Jiaotong University, Chengdu 611756, China; 2 Peng Cheng Laboratory, Shenzhen 518055, China; 3 Department of Electronics, Peking University, Beijing 100871, China

**Keywords:** differential privacy, distributed machine learning, mutual information, Gaussian noise, trade-off

## Abstract

In distributed machine learning (DML), though clients’ data are not directly transmitted to the server for model training, attackers can obtain the sensitive information of clients by analyzing the local gradient parameters uploaded by clients. For this case, we use the differential privacy (DP) mechanism to protect the clients’ local parameters. In this paper, from an information-theoretic point of view, we study the utility–privacy trade-off in DML with the help of the DP mechanism. Specifically, three cases including independent clients’ local parameters with independent DP noise, dependent clients’ local parameters with independent/dependent DP noise are considered. Mutual information and conditional mutual information are used to characterize utility and privacy, respectively. First, we show the relationship between utility and privacy for the three cases. Then, we show the optimal noise variance that achieves the maximal utility under a certain level of privacy. Finally, the results of this paper are further illustrated by numerical results.

## 1. Introduction

With the rapid development of data-driven intelligent applications and the increasing attention on data security, distributed machine learning (DML) has been one of the hottest research fields in machine learning. The goal of DML is to deploy tasks with a huge quantity of data and computations to multiple machines in a distributed way, so as to improve the speed and scalability of data computation, reduce the time consumption of tasks, and improve the privacy performance. In Ref. [1], the authors summarized the design principles of a DML platform and algorithm from four aspects: program deployment and execution, task communication mode, and communication content. In Ref. [2], the authors analyzed and summarized the research status of machine learning algorithms and parallel algorithms based on big data. In Ref. [3], the authors compared the scale and availability of the current mainstream DML platforms, analyzed the fault tolerance and bottlenecks of these platforms, and compared their effects on handwritten data sets. In Ref. [4], the authors reviewed the research status and application of parallel machine learning algorithms, and looked forward to its development trend. In Ref. [5], the authors reviewed some popular algorithms and optimization techniques in the field of machine learning, and focused on the current status, applications, and future development trends of related platforms and algorithms for DML.

However, with the problem of privacy leakage caused by data sharing in DML, researchers have tried to study protection schemes in DML. Specifically, Ref. [6] considered the privacy protection in DML in the case of arbitrary worker collusion. Random quantization was used to convert the data set and weight vector of each round into a finite field, and Lagrange coding was used to encode the quantized value and random matrix to protect privacy. Subsequently, Ref. [7] proposed a DML framework for privacy protection, which eliminated the assumption of trusted servers and provided users with differentiated privacy according to the sensitivity of data and the trust of servers. In addition, Ref. [8] proposed a distributed learning algorithm based on differential privacy, which kept both the data and the model information theoretically private, while allowing an efficient parallelization of training across distributed workers. Recently, Ref. [9] compared the impact of two different privacy protection methods, local differential privacy and federated learning, on DML. The results showed that differential privacy could achieve the best misclassification rate below 20 percent. To sum up, existing discussions about data privacy in DML mainly focus on Lagrange coding, differential privacy, and federated learning. Since differential privacy is promising in the privacy preserving of DML, in this paper, we choose a differential privacy mechanism as our main tool for analyzing the properties of distributed machine learning systems.

In differential privacy, data need to be added by noise to ensure data privacy. Utility is used to characterize the usefulness of the polluted data generated by applying differential privacy to the original data. From an information theory aspect, mutual information characterizes the correlation between two random variables, and [10] used the mutual information as a way to characterize the utility of the polluted data generated by differential privacy. Hence, in this paper, we also used mutual information to define utility in a differential privacy mechanism.

In order to avoid reverse data retrieval in DML, the differential privacy mechanism was introduced to add noise to the parameters uploaded by clients, which protects the privacy of each client data. DP is a privacy protection mechanism proposed by Dwork et al. [11,12,13,14,15,16]. This mechanism uses random noise to ensure that the public output does not leak the client’s privacy. The kinds of added noise generally include Laplacian noise [6], Gaussian noise [17], and exponential noise [18]. However, among the differential privacy mechanism studies, most of them focus on how to reduce the amount of privacy leakage and ignore the utility of the data after noise addition. There is little literature on the relationship between the utility and privacy of the data after noise addition. For example, Ref. [19] used the minimum entropy to quantify the amount of information leakage and calculated the upper bound of information leakage $ulog_{2}\frac{ve^{\epsilon }}{v-1+e^{\epsilon }}$ when the DP conditions were satisfied. Ref. [20] and others defined a formula for information privacy by defining the posterior probability of the same query result of adjacent data sets and proved that if a mechanism satisfies the information privacy with the security parameter, then it also satisfied the differential privacy 2ϵ, and proved the upper limit $\frac{\epsilon }{2n}$ of mutual information between the data sets and the query return value. In [21], the authors proved that in the joint differential privacy of two data sets, the upper bound of mutual information between data sets and query results was further reduced, and the maximum value was 3nϵ. In [22], the authors studied the boundary between the maximum allowable distortion and the privacy budget in the case of noninteractive data release. At the same time, they compared the privacy protection strength of differential privacy with that of reconfigurable privacy and mutual information privacy under the same distortion. The degree of distortion could directly measure the utility of the algorithm mechanism. The optimization problem was established in the paper, which solved the problem of the maximum degree of distortion of different privacy protection mechanisms under the condition of satisfying differential privacy.

As is known to all, utility is one of the important indicators to measure the performance of algorithms in DP. Hence, we aim to find the utility–privacy trade-off of DML from an information-theoretic point of view in this paper. Specifically, three cases including independent clients’ local parameters with independent DP noise and dependent clients’ local parameters with independent/dependent DP noise are considered. We assume that the local parameters and added noise in distributed machine learning are subject to a Gaussian distribution. This is because Gaussian distribution models are widely used in machine learning. Many machine-learning models with probability distribution as the core mostly assume that the data have Gaussian distributions, e.g., logistic regression models, naive Bayes models, and so on. Why can some data be assumed to follow a Gaussian distribution? The intuitive reason is that real-life examples generally satisfy Gaussian distribution, such as the distribution of students’ grades. Furthermore, a Gaussian distribution has many advantages: (1) It is easy to describe, and only two parameters are needed to describe it, the mean and variance, which are the essential information of the distribution. (2) A Gaussian distribution is easy to calculate. It has some good mathematical properties. The data that obey a Gaussian distribution still obey a Gaussian distribution after some operations. For example, a linear combination of normal random variables is still a normal random variable. (3) Many random variables in reality are formed by the combined influence of a large number of independent random factors, and each of the individual factors plays a small role in the overall impact. Such random variables tend to approximately obey a Gaussian distribution (objective background to the central limit theorem). (4) When the mean and variance are known, the entropy of the Gaussian distribution is the largest among all distributions. When the data distribution is unknown, the model with the largest entropy is usually selected. Therefore, it is reasonable to assume that the local parameters and the added noise in our distributed machine learning follow a Gaussian distribution.

Based on the above three cases, the main research methods of this paper are as follows. First, we establish the utility–privacy trade-off for these three cases. Then, we determine the optimum noise variances that achieve the maximal utility under a certain level of privacy. Finally, we further explain the results of this paper by numerical examples.

The remainder of this paper is organized as follows. Section 2 mainly introduces the background knowledge of DML and DP, gives the framework of DML–DP established in this paper, and uses mutual information and conditional mutual information to characterize utility and privacy. Section 3 analyzes the relationship between utility and privacy in DML based on the DP framework and gives the noise level that can obtain the maximum utility under the condition of privacy with three different cases, including independent clients’ local parameters with independent DP noise and dependent clients’ local parameters with independent/dependent DP noise. Section 4 summarizes all the results and discusses the limitations of this paper and future work.

## 2. Preliminaries and Model Formulation

In this section, the preliminary background knowledge of DML and DP is introduced. In addition, we present the distributed machine learning–(mutual information-differential privacy) (DML–(MI-DP)) model that is discussed in the next section.

### 2.1. Preliminaries

*Distributed machine learning*: The goal of DML is to solve how to coordinate and utilize a large number of GPU clusters and massive data to complete the training of a deep learning model and obtain good convergence, so as to achieve relatively high performance. DML involves how to allocate training tasks, how to allocate computing resources, and coordinate various functional modules to achieve the balance between training speed and accuracy. A DML system usually includes the following main modules: data model partition module, single machine optimization module, communication module, and model and data aggregation module. Each module has a variety of implementations, and each implementation method can also be arranged and combined, which makes the methods of DML diverse.

**Figure 1:**
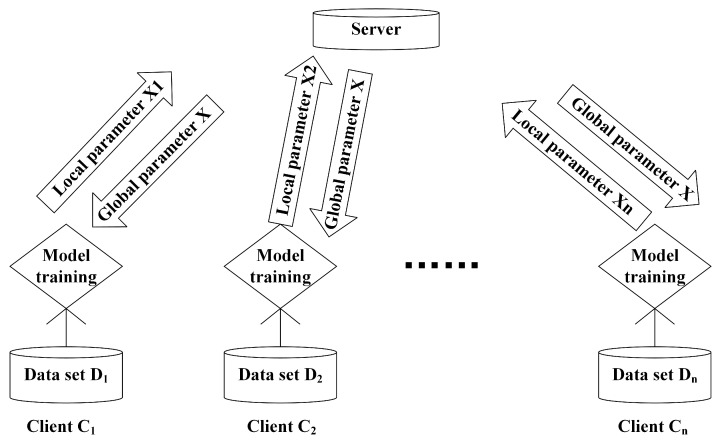
A general distributed machine learning framework.

In this paper, we mainly studied the privacy disclosure problem from the DML framework of data partitioning. In order to visualize the problems studied, this paper adopts the following framework, as shown in Figure 1. The main learning process is as follows: There is a central server and *n* clients. An active client inputs the locally owned data set into the model, and after the model is trained, the model parameter is obtained by the client and then uploaded to the server. On the server side, after it has received the model parameters (also called local parameters) provided by each client, it integrates the local parameters into a global parameter in some way. Here, note that $D_{i}$ represents the data set owned by the client $C_{i}$, and $X_{i}$, i∈{1,2,…,n} is the gradient variable of the model trained locally.

*Differential privacy*: DP prevents differential attacks. The goal of DP is to protect the privacy of each entry in the database while answering queries about the total quantity of data. There are several definitions of DP, such as traditional DP [23] and Renyi DP [24,25,26]. Since Shannon’s definition of mutual information has been widely adopted in DP, we also used Shannon’s definition of mutual information in differential privacy (MI-DP) in this paper. In [10], the authors proposed the concept of MI-DP by defining similarity and demonstrated the relationship between MI-DP and the two types of traditional DP in terms of security strength. In fact, the MI-DP is sandwiched between ϵ-differential privacy and (ϵ, δ)-differential privacy in terms of its strength [27]. MI-DP is fundamentally related to conditional mutual information. The conceptual advantage of using mutual information, aside from yielding a simpler and more intuitive definition of differential privacy, is that its properties are well understood. Several properties of differential privacy are easily verified for the conditional mutual information [27].

**Definition** **1** (Mutual-Information Differential Privacy [10]). *A randomized mechanism $P_{R|M^{n}}$ satisfies ϵ-mutual-information differential privacy if*

(1) $$\begin{array}{c} \max_{\begin{array}{c} i \end{array}}I\left(M_{i};R|M^{-i}\right)\leq \epsilon , \end{array}$$

*where R is the output of randomized mechanism $P_{R|M^{n}}$, $M^{n}=\left(M_{1},\ldots ,M_{n}\right)$ is a database, $M^{-i}$ denotes the other data in the database except for the $M_{i}$ element, and ϵ>0 represents the privacy budget: the larger ϵ, the lower the privacy requirements, and the smaller ϵ, the stronger the privacy.*

This definition clearly reveals what kind of privacy is guaranteed by DP and what kind of privacy is not, which is easy to understand intuitively. For example, we can suppose that an adversary already knows about all except a certain data element, and they want to use the randomized mechanism to analyze the remaining data information. This is also known as the strong adversary hypothesis. This hypothesis is clearly revealed in MI-DP by conditional mutual information [10].

By adding random noise, DP ensures that the public output results will not be significantly changed due to an entity being in the data set and gives a quantitative model for the degree of privacy leakage. Different kinds of noise can be added to this model. For example, Laplace noise, exponential noise, or Gaussian noise can be chosen.

### 2.2. Model Formulation

**Figure 2:**
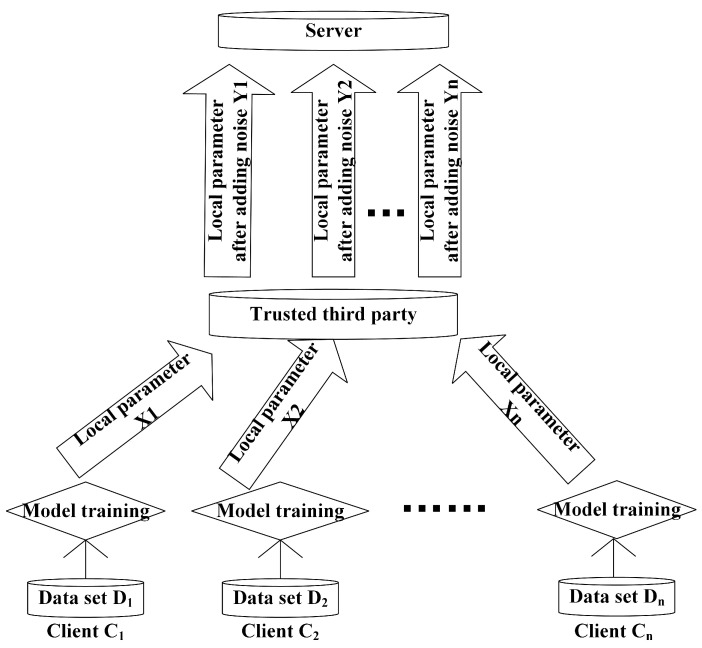
The general distributed machine learning framework based on differential privacy.

Our framework, a general distributed machine learning framework based on differential privacy: DML can train large quantities of data locally due to its distributed structure. However, in the process of DML, an attacker can analyze the model parameters $X_{i}$ (i∈{1,2,…,n}) uploaded by each client to obtain the client’s sensitive information [28,29]. Therefore, we use the method of combining DP with Gaussian noise and distributed machine learning to deal with the risk of such privacy leakage. The model architecture is shown in Figure 2. In fact, it adds random noise on the basis of the general DML framework to complete DP. After the clients have trained the model locally, the model parameters $X_{i}$ (i∈{1,2,…,n}) are not directly uploaded to the server, but are handed over to a trusted third party. We assume that the channel through which the clients transmit the local parameters to the trusted third party is absolutely safe and reliable. The third party adds random Gaussian noise $Z_{i}$ to each local parameter $X_{i}$ (i∈{1,2,…,n}), and finally, after adding noise, the local parameters are transmitted to the server by a trusted third party to complete the aggregation and obtain the global parameter.

**Figure 3:**
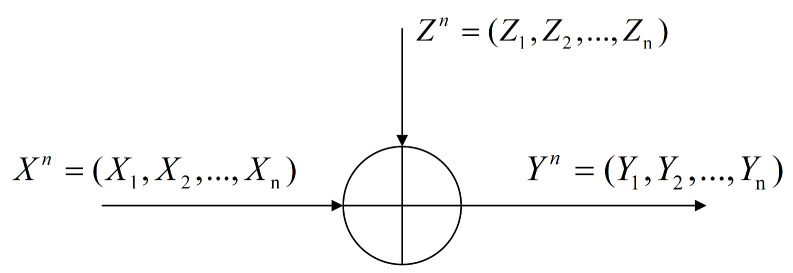
The relationship between $X^{n}$, $Z^{n}$ and $Y^{n}$.

In this model, the key step is to design the noise $Z_{i}$ (i∈{1,2,…,n}), which should not only meet the MI-DP condition, but also maximize the utility of local parameters after adding noise. The relationship between $X^{n}$, $Z^{n}$ and $Y^{n}$ is represented by Figure 3,

(2) $$\begin{array}{c} Y^{n}=X^{n}+Z^{n}. \end{array}$$

Here, note that $X^{n}=\left(X_{1},\ldots ,X_{n}\right)$, $Z^{n}=\left(Z_{1},\ldots ,Z_{n}\right)$, $Y^{n}=\left(Y_{1},\ldots ,Y_{n}\right)$, $X^{n}$ is the set of local parameters obtained from the local training model of all clients, and $Z^{n}$ is the set of Gaussian random noise added to the local parameters. $Y^{n}$ is the set of all local parameters after adding noise.

Through the definition of MI-DP, we know that $X_{i}$, $Z_{i}$ and $Y_{i}$ (i∈{1,2,…,n}) must meet the following condition:

(3) $$\begin{array}{c} \max_{\begin{array}{c} i \end{array}}I\left(X_{i};Y^{n}|X^{-i}\right)\leq \epsilon , \end{array}$$

where $X^{-i}=\left(X_{1},\ldots ,X_{i-1},X_{i+1},\ldots ,X_{n}\right)$. In this paper, we regard the left side of (3) as the expression of satisfying privacy.

By the meaning of mutual information, the expression that measures the utility of the noisy local parameters is denoted by,

(4) $$\begin{array}{c} U=\frac{I(X^{n};Y^{n})}{n}, \end{array}$$

which is regarded as the expression of utility.

In the next section, we mainly introduce the two important tasks of this paper: The first part focuses on how to design the variance of the noise to make the noisy local parameters have the best utility (the maximum value of (4) while satisfying the Equation (3)). The second part focuses on exploring the relationship between the utility and privacy of the noisy local parameters, in other words, the relationship between *U* and ϵ.

## 3. Analysis of the Amount of Noise Added and Exploration of the Relationship between Utility and Privacy

In this section, the local parameter $X_{i}$ (i∈{1,2,…,n}) obtained by the client’s local training is a Gaussian random variable, and the added noise $Z_{i}$ (i∈{1,2,…,n}) is also a random Gaussian variable. We used the conditional expression of privacy and the expression of utility so that the most suitable noise variance could be designed after calculation, and the designed noise could not only meet the definition of DP, but also optimize the utility of noisy local parameters.

Therefore, this problem could be mathematized and expressed as: How should the variance of the added noise be designed to make $U=\frac{I(X^{n};Y^{n})}{n}$ maximum under the conditions $\max_{\begin{array}{c} i \end{array}}I\left(X_{i};Y^{n}|X^{-i}\right)\leq \epsilon$? The three cases are described below:

Case 1: independent noise added to the independent local parameters.

Case 2: independent noise added to the dependent local parameters.

Case 3: dependent noise added to the dependent local parameters.

After solving this problem, the most suitable noise variances were designed. Next, we studied theoretically the relationship between the utility and privacy of the noisy local parameters in the DML based on the DP framework.

### 3.1. Case 1: Independent Noise Added to Independent Local Parameters

In Case 1, we assumed that the parameters of each client were independent of each other. Consequently, this case corresponded to the actual application scenario, which could be used for training data with little or no correlation between them, e.g., word recognition, spam classification, etc.

The clients’ local parameters $X_{i}∼N\left(0,\sigma_{i}^{2}\right)$ were independent of each other, where ∼ denoted “distributed as”, and $\sigma_{i}^{2}$ varied with *i* (i∈{1,2,…,n}), that is to say, the distribution of local parameters of each client was different. The noise added to each local parameter was also independent and is distributed as $Z_{i}\;∼N\left(0,\sigma^{2}\right)$.

**Theorem** **1.** *The optimum Gaussian noise variance $\sigma^{2}$ in Case 1 is given by*

$$\sigma^{2}=\frac{\sigma_{xmax}^{2}}{2^{2\epsilon }-1},$$

*where $\sigma_{xmax}^{2}=\max\left\{\sigma_{1}^{2},\ldots ,\sigma_{n}^{2}\right\}$, and $\sigma^{2}$ achieves the maximal utility $U=\frac{I(X^{n};Y^{n})}{n}$ under a certain secrecy level $\max_{\begin{array}{c} i \end{array}}I\left(X_{i};Y^{n}|X^{-i}\right)\leq \epsilon$.*

**Proof of Theorem** **1.** Since the relationship between $Y_{i}$, $X_{i}$ and $Z_{i}$ is $Y_{i}=X_{i}+Z_{i}$ (i∈{1,2,…,n}), we easily obtain that $Y_{i}$ are independent of each other and distributed as $Y_{i}\;∼N\left(0,\sigma_{i}^{2}+\sigma^{2}\right)$. From the definition of (3), we have

(5) $$\begin{array}{ccc} & & I(X_{i};Y^{n}|X^{-i})\\ & & =h\left(Y^{n}|X^{-i}\right)-h\left(Y^{n}|X^{n}\right)\\ & & =h\left(X_{i}+Z_{i},Z^{-i}\right))-h\left(Z^{n}\right)\\ & & =\frac{1}{2}\log\left[{\left(2\pi e\right)}^{n}\left(\sigma_{i}^{2}+\sigma^{2}\right)\sigma^{2(n-1)}\right]-\frac{1}{2}\log\left[{\left(2\pi e\right)}^{n}\sigma^{2n}\right]\\ & & =\frac{1}{2}\log\left(1+\frac{\sigma_{i}^{2}}{\sigma^{2}}\right)\leq \epsilon . \end{array}$$

From (5) we deduce that the variance $\sigma^{2}$ of the designed noise $Z_{i}$ should satisfy the following inequality

(6) $$\begin{array}{c} \sigma^{2}\geq \frac{\sigma_{xmax}^{2}}{2^{2\epsilon }-1}, \end{array}$$

where $\sigma_{xmax}^{2}=\max\left\{\sigma_{1}^{2},\ldots ,\sigma_{n}^{2}\right\}$. After clarifying the value range of the noise variance under the privacy condition, the next step is to select the most suitable variance value in the value range to get the best utility (make $U=\frac{I(X^{n};Y^{n})}{n}$ maximal). From (4), we have

(7) $$\begin{array}{ccc} & & U=\frac{1}{n}I\left(X^{n};Y^{n}\right)\\ & & =\frac{1}{n}\left[h\left(Y^{n}\right)-h\left(Y^{n}|X^{n}\right)\right]\\ & & =\frac{1}{n}\left[h\left(Y^{n}\right)-h\left(Z^{n}\right)\right]\\ & & =\frac{1}{2n}\log\left[{\left(2\pi e\right)}^{n}\left(\sigma_{1}^{2}+\sigma^{2}\right)*\ldots *\left(\sigma_{n}^{2}+\sigma^{2}\right)\right]\\ & & -\frac{1}{2n}\log\left[{\left(2\pi e\right)}^{n}\sigma^{2n}\right]\\ & & =\frac{1}{2n}\sum_{i=1}^{n}\log\left(1+\frac{{\sigma_{i}}^{2}}{\sigma^{2}}\right). \end{array}$$

From (8), it can be clearly observed that $U=\frac{I(X^{n};Y^{n})}{n}$ is a monotonically decreasing function of $\sigma^{2}$. Therefore, when $\sigma^{2}$ takes the minimum value

(8) $$\begin{array}{c} \sigma^{2}=\frac{\sigma_{xmax}^{2}}{2^{2\epsilon }-1}, \end{array}$$

the expression of utility U takes the maximum value

(9) $$\begin{array}{c} U_{max}=\frac{1}{2n}\sum_{i=1}^{n}\log\left[1+\frac{{\sigma_{i}}^{2}\left(2^{2\epsilon }-1\right)}{\sigma_{xmax}^{2}}\right]. \end{array}$$

□

In summary, the problem of how to design the size of the noise variance in Case 1 was solved and the first part of the work of Case 1 completed.

After obtaining the optimum noise variance, the next step was to study the relationship between utility and privacy in Case 1 (that is, the relationship between $U_{max}$ and ϵ), where (9) is the functional relationship between $U_{max}$ and ϵ.

**Figure 4:**
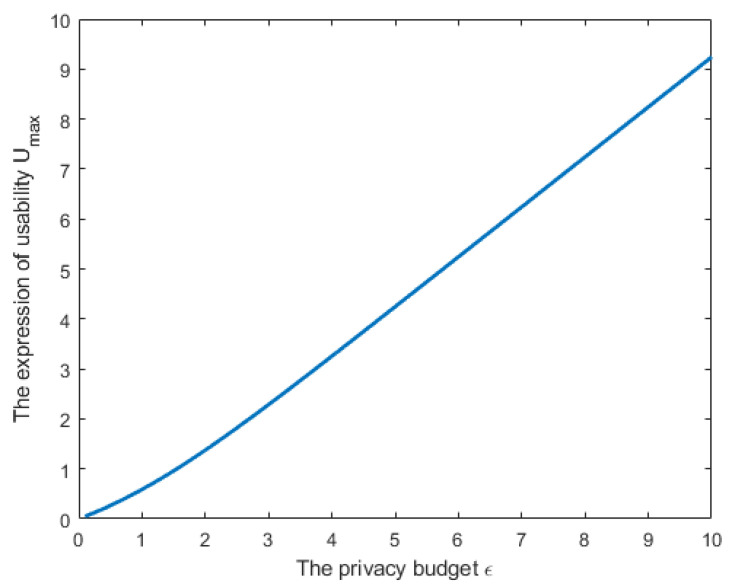
The relationship between utility and privacy in Case 1.

Figure 4 plots the relationship between $U_{max}$ and ϵ based on (9). In this figure, we assumed that n=101, $\sigma_{i}^{2}$ (i∈{1,2,…,101}) to be a random value between 0 and 1. It can be seen from the figure that when the local parameters of clients are independent of each other, and the noise is also designed to be independent, the amounts of the privacy budget and utility are proportional. The larger the privacy budget value (the greater the risk of privacy leakage), the bigger the value of utility.

### 3.2. Case 2: Independent Noise Added to Dependent Local Parameters

Case 2 was different from Case 1, as we assumed that the clients’ local parameters were dependent of each other. This case is used for practical application scenarios of correlation between training data. For example, machine learning can be applied to explore the impact of age on health in humans. It is well known that there is a certain correlation between age and human health indicators.

The clients’ local parameters $X_{i}\;∼N\left(0,\sigma_{m}^{2}\right)$ were dependent of each other. When i≠j, $E\left[X_{i}X_{j}\right]=\sigma_{k}^{2}$ (j∈{1,2,…,n}). The distributions of local parameters of each client were the same. The noise added to each local parameter $Z_{i}\;∼N\left(0,\sigma^{2}\right)$ (i∈{1,2,…,n}) was independent.

**Theorem** **2.** *The optimum Gaussian noise variance in Case 2 is given by*

$$\sigma^{2}=\frac{\sigma_{m}^{2}}{2^{2\epsilon }-1}+\frac{\left(n-1\right)\sigma_{k}^{4}}{\left(2^{2\epsilon }-1\right)\left[\sigma_{m}^{2}+\left(n-2\right)\sigma_{k}^{2}\right]},$$

*which achieves the maximal utility $U=\frac{I(X^{n};Y^{n})}{n}$ under a certain secrecy level $\max_{\begin{array}{c} i \end{array}}I\left(X_{i};Y^{n}|X^{-i}\right)\leq \epsilon$.*

**Proof of Theorem** **2.** Since $Y_{i}=X_{i}+Z_{i}$ (i∈{1,2,…,n}) and $X_{i}$ are dependent of each other, $Y_{i}\;∼N\left(0,\sigma_{m}^{2}+\sigma^{2}\right)$ are dependent of each other. We assume that when i≠j, $E\left[Y_{i}Y_{j}\right]=\sigma_{k}^{2}$ (j∈{1,2,…,n}). We put the value of each variable into the expression of satisfying privacy, and it is calculated as

(10) $$\begin{array}{ccc} & & I(X_{i};Y^{n}|X^{-i})\\ & & =h\left(Y^{n}|X^{-i}\right)-h\left(Y^{n}|X^{n}\right)\\ & & =h\left(X_{i}+Z_{i},Z^{-i}|X^{-i}\right)-h\left(Z^{n}\right)\\ & & =h\left(X_{i}+Z_{i}|X^{-i}\right)+h\left(Z^{-i}\right)-h\left(Z^{n}\right)\\ & & =h\left(X_{i}+Z_{i},X^{-i}\right)-h\left(X^{-i}\right)+h\left(Z_{i}\right)\\ & & =\frac{1}{2}{\log[\left(2\pi e\right)}^{n}|COV\left(X_{i}+Z_{i},X^{-i}\right)\left|\right]-\frac{1}{2}\log[{\left(2\pi e\right)}^{n-1}|COV\left(X^{-i}\right)\left|\right]-\frac{1}{2}\log\left(2\pi e\sigma^{2}\right)\\ & & =\frac{1}{2}\log\left(\frac{\left|COV(X_{i}+Z_{i},X^{-i})\right|}{|COV\left(X^{-i}\right)|\sigma^{2}}\right)\leq \epsilon . \end{array}$$

for $\left|COV(X_{i}+Z_{i},X^{-i})\right|$ and $\left|COV(X^{-i})\right|$, we have

(11) $$\begin{array}{ccc} & & \left|COV(X_{i}+Z_{i},X^{-i})\right|\\ & & ={\left|\begin{array}{cccc} E{\left(X_{i}+Z_{i}\right)}^{2} & EX_{i}X_{1} & \cdots & EX_{i}X_{n}\\ EX_{1}X_{i} & EX_{1}^{2} & \cdots & EX_{1}X_{n}\\ \vdots & \vdots & \ddots & \vdots \\ EX_{n}X_{i} & EX_{n}X_{1} & \cdots & EX_{n}^{2} \end{array}\right|}_{n\times n}\\ & & ={\left|\begin{array}{cccc} \sigma_{m}^{2}+\sigma^{2} & \sigma_{k}^{2} & \cdots & \sigma_{k}^{2}\\ \sigma_{k}^{2} & \sigma_{m}^{2} & \cdots & \sigma_{k}^{2}\\ \vdots & \vdots & \ddots & \vdots \\ \sigma_{k}^{2} & \sigma_{k}^{2} & \cdots & \sigma_{m}^{2} \end{array}\right|}_{n\times n}\\ & & ={\left(\sigma_{m}^{2}-\sigma_{k}^{2}\right)}^{n-1}\left(\sigma_{m}^{2}+\sigma^{2}\right)-\left(n-1\right)\left(\sigma_{k}^{2}-\sigma_{m}^{2}-\sigma^{2}\right)\sigma_{k}^{2}{\left(\sigma_{m}^{2}-\sigma_{k}^{2}\right)}^{n-2}, \end{array}$$

(12) $$\begin{array}{ccc} & & \left|COV(X^{-i})\right|\\ & & ={\left|\begin{array}{cccc} EX_{1}^{2} & EX_{1}X_{2} & \cdots & EX_{1}X_{n}\\ EX_{2}X_{1} & EX_{2}^{2} & \cdots & EX_{2}X_{n}\\ \vdots & \vdots & \ddots & \vdots \\ EX_{n}X_{1} & EX_{n}X_{2} & \cdots & EX_{n}^{2} \end{array}\right|}_{\left(n-1)\times (n-1\right)}\\ & & ={\left|\begin{array}{cccc} \sigma_{m}^{2} & \sigma_{k}^{2} & \cdots & \sigma_{k}^{2}\\ \sigma_{k}^{2} & \sigma_{m}^{2} & \cdots & \sigma_{k}^{2}\\ \vdots & \vdots & \ddots & \vdots \\ \sigma_{k}^{2} & \sigma_{k}^{2} & \cdots & \sigma_{m}^{2} \end{array}\right|}_{\left(n-1)\times (n-1\right)}\\ & & =\left[\sigma_{m}^{2}+\left(n-2\right)\sigma_{k}^{2}\right]{\left(\sigma_{m}^{2}-\sigma_{k}^{2}\right)}^{n-2}. \end{array}$$

According to (11), (12) and (10) can be expressed as

(13) $$\begin{array}{ccc} & & I(X_{i};Y^{n}|X^{-i})\\ & & =\frac{1}{2}\log\left(\frac{{\left(\sigma_{m}^{2}-\sigma_{k}^{2}\right)}^{n-1}\left(\sigma_{m}^{2}+\sigma^{2}\right)}{\left[\sigma_{m}^{2}+\left(n-2\right)\sigma_{k}^{2}\right]{\left(\sigma_{m}^{2}-\sigma_{k}^{2}\right)}^{n-2}\sigma^{2}}-\frac{\left(n-1\right)\left(\sigma_{k}^{2}-\sigma_{m}^{2}-\sigma^{2}\right)\sigma_{k}^{2}{\left(\sigma_{m}^{2}-\sigma_{k}^{2}\right)}^{n-2}}{\left[\sigma_{m}^{2}+\left(n-2\right)\sigma_{k}^{2}\right]{\left(\sigma_{m}^{2}-\sigma_{k}^{2}\right)}^{n-2}\sigma^{2}}\right)\\ & & \leq \epsilon . \end{array}$$

From (13), we deduce that the variance $\sigma^{2}$ of the designed noise $Z_{i}$ should satisfy the following inequality

(14) $$\begin{array}{c} \sigma^{2}\geq \frac{\sigma_{m}^{2}}{2^{2\epsilon }-1}+\frac{\left(n-1\right)\sigma_{k}^{4}}{\left(2^{2\epsilon }-1\right)\left[\sigma_{m}^{2}+\left(n-2\right)\sigma_{k}^{2}\right]}. \end{array}$$

After clarifying the value range of the noise variance under the privacy condition, the next step is to select the most suitable variance value in the value range to get the best utility (make $U=\frac{I(X^{n};Y^{n})}{n}$ maximal).

(15) $$\begin{array}{ccc} & & U=\frac{1}{n}I\left(X^{n};Y^{n}\right)\\ & & =\frac{1}{n}\left[h\left(Y^{n}\right)-h\left(Y^{n}|X^{n}\right)\right]\\ & & =\frac{1}{n}\left[h\left(Y^{n}\right)-h\left(Z^{n}\right)\right]\\ & & =\frac{1}{2n}{\log[\left(2\pi e\right)}^{n}|COV\left(Y_{1},\ldots ,Y_{n}\right)\left|\right]-\frac{1}{2n}\log\left[{\left(2\pi e\right)}^{n}\sigma^{2n}\right]\\ & & =\frac{1}{2n}\log\left(\frac{\left|COV(Y_{1},\ldots ,Y_{n})\right|}{\sigma^{2n}}\right). \end{array}$$

For $\left|COV(Y_{1},\ldots ,Y_{n})\right|$, we have

(16) $$\begin{array}{ccc} & & \left|COV(Y_{1},\ldots ,Y_{n})\right|\\ & & ={\left|\begin{array}{cccc} E{\left(Y_{1}\right)}^{2} & EY_{1}Y_{2} & \cdots & EY_{1}Y_{n}\\ EY_{2}Y_{n} & EY_{2}^{2} & \cdots & EY_{2}Y_{n}\\ \vdots & \vdots & \ddots & \vdots \\ EY_{n}Y_{1} & EY_{n}Y_{2} & \cdots & EY_{n}^{2} \end{array}\right|}_{n\times n}\\ & & ={\left|\begin{array}{cccc} \sigma_{m}^{2}+\sigma^{2} & \sigma_{k}^{2} & \cdots & \sigma_{k}^{2}\\ \sigma_{k}^{2} & \sigma_{m}^{2}+\sigma^{2} & \cdots & \sigma_{k}^{2}\\ \vdots & \vdots & \ddots & \vdots \\ \sigma_{k}^{2} & \sigma_{k}^{2} & \cdots & \sigma_{m}^{2}+\sigma^{2} \end{array}\right|}_{n\times n}\\ & & =\left[\sigma_{m}^{2}+\sigma^{2}+\left(n-1\right)\sigma_{k}^{2}\right]{\left(\sigma_{m}^{2}+\sigma^{2}-\sigma_{k}^{2}\right)}^{n-1}, \end{array}$$

From (16) and (15), this can be expressed as

(17) $$\begin{array}{ccc} & & U=\frac{1}{n}I\left(X^{n};Y^{n}\right)\\ & & =\frac{1}{2n}\log\left(\frac{\sigma_{m}^{2}+\sigma^{2}+\left(n-1\right)\sigma_{k}^{2}}{\sigma^{2n}}\right)+\frac{1}{2n}\log\left(\frac{{\left(\sigma_{m}^{2}+\sigma^{2}-\sigma_{k}^{2}\right)}^{n-1}}{\sigma^{2n}}\right). \end{array}$$

From (17), it can be clearly observed that $U=\frac{I(X^{n};Y^{n})}{n}$ is a monotonically decreasing function of $\sigma^{2}$. Therefore, when $\sigma^{2}$ takes its minimum value ($\sigma^{2}=\frac{\sigma_{m}^{2}}{2^{2\epsilon }-1}+\frac{\left(n-1\right)\sigma_{k}^{4}}{\left(2^{2\epsilon }-1\right)\left[\sigma_{m}^{2}+\left(n-2\right)\sigma_{k}^{2}\right]}$ is denoted as $\sigma_{min}^{2}$), the expression of utility U takes its maximum value

(18) $$\begin{array}{ccc} & & U_{max}=\frac{1}{2n}\log\left[\sigma_{m}^{2}+\sigma_{min}^{2}+\left(n-1\right)\sigma_{k}^{2}\right]+\frac{1}{2n}\log\left(\frac{{\left(\sigma_{m}^{2}+\sigma_{min}^{2}-\sigma_{k}^{2}\right)}^{n-1}}{\sigma_{min}^{2n}}\right). \end{array}$$

□

In summary, the problem of how to design the size of the noise variance in Case 2 was solved and the first part of the work of Case 2 completed.

Next, we carried out the second part of the work: After obtaining the optimal noise variance, the next step was to study the relationship between utility and privacy in Case 2 (the relationship between $U_{max}$ and ϵ), where (18) is the functional relationship between $U_{max}$ and ϵ.

**Figure 5:**
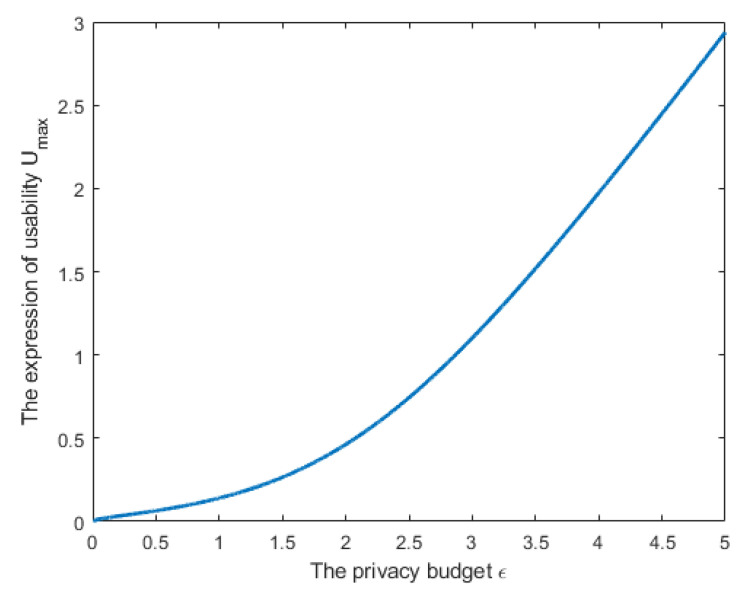
The relationship between utility and privacy in Case 2.

Figure 5 plots the relationship between $U_{max}$ and ϵ based on (18). In this figure, we assumed that $\sigma_{m}^{2}=2$, $\sigma_{k}^{2}=1.8$, and n=101. It can be seen from the figure that when the local parameters of clients are dependent of each other, and the added noise is designed to be independent, the amounts of the privacy budget and utility are proportional. We conclude that the larger the privacy budget value (the greater the risk of privacy leakage), the bigger the value of utility.

### 3.3. Case 3: Dependent Noise Added to Local Parameters

Case 3 was different from Case 2, as we assumed that the noise added to each local parameter was dependent. In order to check whether dependent noise performed better than independent noise, we studied Case 3. The application scenario of Case 3 is still that the parameters are correlated, for example, the study of the correlation between human lifespan and gender.

In Case 3, the noise added to each local parameter $Z_{i}\;∼N\left(0,\sigma^{2}\right)$ (i∈{1,2,…,n}) was dependent. When i≠j, $E\left[Z_{i}Z_{j}\right]=\sigma_{e}^{2}$, j∈{1,2,…,n}. The clients’ local parameters $X_{i}\;∼N\left(0,\sigma_{m}^{2}\right)$ were also dependent of each other. When i≠j, $E\left[X_{i}X_{j}\right]=\sigma_{k}^{2}$, j∈{1,2,…,n}. The distributions of local parameters of each client were the same.

For the problem to be solved, we made the following analysis. Case 3 was different from Cases 1 and 2. There were two noise parameters in Case 3. We had to design the most suitable noise to make $U=\frac{I(X^{n};Y^{n})}{n}$ maximum under the conditions $\max_{\begin{array}{c} i \end{array}}I\left(X_{i};Y^{n}|X^{-i}\right)\leq \epsilon$. Thus, the computational difficulty was also much higher than in Cases 1 and 2. As we know, $Y_{i}=X_{i}+Z_{i}$, i∈{1,2,…,n}, so $Y_{i}\;∼N\left(0,\sigma_{m}^{2}+\sigma^{2}\right)$ are dependent of each other. We assumed that when i≠j, $E\left[Y_{i}Y_{j}\right]=\sigma_{k}^{2}+\sigma_{e}^{2}$, $E\left[X_{i}Y_{j}\right]=\sigma_{k}^{2}$, j∈{1,2,…,n}. We put the value of each variable into the expression of satisfying privacy, which could be calculated as

(19) $$\begin{array}{ccc} & & I(X_{i};Y^{n}|X^{-i})\\ & & =h\left(Y^{n}|X^{-i}\right)-h\left(Y^{n}|X^{n}\right)\\ & & =h\left(Y^{n},X^{-i}\right)-h\left(X^{-i}\right)-h\left(Z^{n}\right)\\ & & =\frac{1}{2}{\log[\left(2\pi e\right)}^{2n-1}|COV\left(Y^{n},X^{-i}\right)\left|\right]-\frac{1}{2}\log[{\left(2\pi e\right)}^{n-1}|COV\left(X^{-i}\right)\left|\right]-\frac{1}{2}\log{\left(2\pi e\right)}^{n}|COV\left(Z^{n}\right)\left|\right)\\ & & =\frac{1}{2}\log\left(\frac{\left|COV(Y^{n},X^{-i})\right|}{|COV\left(X^{-i}\right)||COV\left(Z^{n}\right)|}\right)\leq \epsilon . \end{array}$$

**Lemma** **1.** *For $\left|COV(Y^{n},X^{-i})\right|$, even if i∈{1,2,…,n} takes different values, the result of determinant $\left|COV(Y^{n},X^{-i})\right|$ is the same.*

**Proof.**  When i=1,

(20) $$\begin{array}{ccc} & & |COV\left(Y^{n},X^{-i}\right)|=|COV\left(Y^{n},X^{-1}\right)|\\ & & ={\left|\begin{array}{c} \begin{array}{cccccccc} EY_{1}^{2} & EY_{1}Y_{2} & \cdots & EY_{1}Y_{n} & EY_{1}X_{2} & EY_{1}X_{3} & \cdots & EY_{1}X_{n}\\ EY_{2}Y_{1} & EY_{2}^{2} & \cdots & \cdots & EY_{2}X_{2} & EY_{2}X_{3} & \cdots & \cdots \\ \cdots & \cdots & \ddots & \cdots & \cdots & \cdots & \ddots & \cdots \\ EY_{n}Y_{1} & \cdots & \cdots & EY_{n}^{2} & EY_{n}X_{2} & \cdots & \cdots & EY_{n}X_{n}\\ EX_{2}Y_{1} & EX_{2}Y_{2} & \cdots & EX_{2}Y_{n} & EX_{2}^{2} & EX_{2}X_{3} & \cdots & EX_{2}X_{n}\\ EX_{3}Y_{1} & EX_{3}Y_{2} & \cdots & \cdots & EX_{3}X_{2} & EX_{3}^{2} & \cdots & \cdots \\ \cdots & \ddots & \cdots & \cdots & \cdots & \cdots & \ddots & \cdots \\ EX_{n}Y_{1} & \cdots & \cdots & EX_{n}Y_{n} & EX_{n}X_{2} & \cdots & \cdots & EX_{n}^{2} \end{array} \end{array}\right|}_{\left(2n-1)\times (2n-1\right)}\\ & & ={\left|\begin{array}{c} \begin{array}{cccccccc} \sigma_{m}^{2}+\sigma^{2} & \sigma_{k}^{2}+\sigma_{e}^{2} & \cdots & \sigma_{k}^{2}+\sigma_{e}^{2} & \sigma_{k}^{2} & \sigma_{k}^{2} & \cdots & \sigma_{k}^{2}\\ \sigma_{k}^{2}+\sigma_{e}^{2} & \sigma_{m}^{2}+\sigma^{2} & \cdots & \cdots & \sigma_{m}^{2} & \sigma_{k}^{2} & \cdots & \cdots \\ \cdots & \cdots & \ddots & \cdots & \cdots & \cdots & \ddots & \cdots \\ \sigma_{k}^{2}+\sigma_{e}^{2} & \cdots & \cdots & \sigma_{m}^{2}+\sigma^{2} & \sigma_{k}^{2} & \cdots & \cdots & \sigma_{m}^{2}\\ \sigma_{k}^{2} & \sigma_{m}^{2} & \cdots & \sigma_{k}^{2} & \sigma_{m}^{2} & \sigma_{k}^{2} & \cdots & \sigma_{k}^{2}\\ \sigma_{k}^{2} & \sigma_{k}^{2} & \cdots & \cdots & \sigma_{k}^{2} & \sigma_{m}^{2} & \cdots & \cdots \\ \cdots & \ddots & \cdots & \cdots & \cdots & \cdots & \ddots & \cdots \\ \sigma_{k}^{2} & \cdots & \cdots & \sigma_{m}^{2} & \sigma_{k}^{2} & \cdots & \cdots & \sigma_{m}^{2} \end{array} \end{array}\right|}_{\left(2n-1)\times (2n-1\right)}. \end{array}$$

When i=2,

(21) $$\begin{array}{ccc} & & |COV\left(Y^{n},X^{-i}\right)|=|COV\left(Y^{n},X^{-2}\right)|\\ & & ={\left|\begin{array}{c} \begin{array}{cccccccc} EY_{1}^{2} & EY_{1}Y_{2} & \cdots & EY_{1}Y_{n} & EY_{1}X_{1} & EY_{1}X_{3} & \cdots & EY_{1}X_{n}\\ EY_{2}Y_{1} & EY_{2}^{2} & \cdots & \cdots & EY_{2}X_{1} & EY_{2}X_{3} & \cdots & \cdots \\ \cdots & \cdots & \ddots & \cdots & \cdots & \cdots & \ddots & \cdots \\ EY_{n}Y_{1} & \cdots & \cdots & EY_{n}^{2} & EY_{n}X_{1} & \cdots & \cdots & EY_{n}X_{n}\\ EX_{1}Y_{1} & EX_{1}Y_{2} & \cdots & EX_{1}Y_{n} & EX_{1}^{2} & EX_{1}X_{3} & \cdots & EX_{1}X_{n}\\ EX_{3}Y_{1} & EX_{3}Y_{2} & \cdots & \cdots & EX_{3}X_{1} & EX_{3}^{2} & \cdots & \cdots \\ \cdots & \ddots & \cdots & \cdots & \cdots & \cdots & \ddots & \cdots \\ EX_{n}Y_{1} & \cdots & \cdots & EX_{n}Y_{n} & EX_{n}X_{1} & \cdots & \cdots & EX_{n}^{2} \end{array} \end{array}\right|}_{\left(2n-1)\times (2n-1\right)}\\ & & ={\left|\begin{array}{c} \begin{array}{cccccccccc} \sigma_{m}^{2}+\sigma^{2} & \sigma_{k}^{2}+\sigma_{e}^{2} & \cdots & \cdots & \sigma_{k}^{2}+\sigma_{e}^{2} & \sigma_{m}^{2} & \sigma_{k}^{2} & \cdots & \cdots & \sigma_{k}^{2}\\ \sigma_{k}^{2}+\sigma_{e}^{2} & \sigma_{m}^{2}+\sigma^{2} & \cdots & \cdots & \cdots & \sigma_{k}^{2} & \sigma_{k}^{2} & \cdots & \cdots & \sigma_{k}^{2}\\ \cdots & \cdots & \ddots & \cdots & \cdots & \sigma_{k}^{2} & \sigma_{m}^{2} & \cdots & \cdots & \sigma_{k}^{2}\\ \cdots & \cdots & \cdots & \ddots & \cdots & \cdots & \cdots & \cdots & \ddots & \cdots \\ \sigma_{k}^{2}+\sigma_{e}^{2} & \cdots & \cdots & \cdots & \sigma_{m}^{2}+\sigma^{2} & \sigma_{k}^{2} & \sigma_{k}^{2} & \cdots & \cdots & \sigma_{m}^{2}\\ \sigma_{m}^{2} & \sigma_{k}^{2} & \sigma_{k}^{2} & \cdots & \sigma_{k}^{2} & \sigma_{m}^{2} & \sigma_{k}^{2} & \cdots & \cdots & \sigma_{k}^{2}\\ \sigma_{k}^{2} & \sigma_{k}^{2} & \sigma_{m}^{2} & \cdots & \sigma_{k}^{2} & \sigma_{k}^{2} & \sigma_{m}^{2} & \cdots & \cdots & \cdots \\ \sigma_{k}^{2} & \sigma_{k}^{2} & \cdots & \ddots & \cdots & \cdots & \cdots & \ddots & \cdots \\ \cdots & \cdots & \cdots & \cdots & \cdots & \cdots & \cdots & \cdots & \ddots & \cdots \\ \sigma_{k}^{2} & \sigma_{k}^{2} & \cdots & \cdots & \sigma_{m}^{2} & \sigma_{k}^{2} & \cdots & \cdots & \cdots & \sigma_{m}^{2} \end{array} \end{array}\right|}_{\left(2n-1)\times (2n-1\right)}\\ & & ={\left(-1\right)}^{2}\left|\begin{array}{c} \begin{array}{cccccccccc} \sigma_{m}^{2}+\sigma^{2} & \sigma_{k}^{2}+\sigma_{e}^{2} & \cdots & \cdots & \sigma_{k}^{2}+\sigma_{e}^{2} & \sigma_{k}^{2} & \sigma_{k}^{2} & \cdots & \cdots & \sigma_{k}^{2}\\ \sigma_{k}^{2}+\sigma_{e}^{2} & \sigma_{m}^{2}+\sigma^{2} & \cdots & \cdots & \cdots & \sigma_{m}^{2} & \sigma_{k}^{2} & \cdots & \cdots & \sigma_{k}^{2}\\ \cdots & \cdots & \ddots & \cdots & \cdots & \sigma_{k}^{2} & \sigma_{m}^{2} & \cdots & \cdots & \sigma_{k}^{2}\\ \cdots & \cdots & \cdots & \ddots & \cdots & \cdots & \cdots & \cdots & \ddots & \cdots \\ \sigma_{k}^{2}+\sigma_{e}^{2} & \cdots & \cdots & \cdots & \sigma_{m}^{2}+\sigma^{2} & \sigma_{k}^{2} & \sigma_{k}^{2} & \cdots & \cdots & \sigma_{m}^{2}\\ \sigma_{k}^{2} & \sigma_{m}^{2} & \sigma_{k}^{2} & \cdots & \sigma_{k}^{2} & \sigma_{m}^{2} & \sigma_{k}^{2} & \cdots & \cdots & \sigma_{k}^{2}\\ \sigma_{k}^{2} & \sigma_{k}^{2} & \sigma_{m}^{2} & \cdots & \sigma_{k}^{2} & \sigma_{k}^{2} & \sigma_{m}^{2} & \cdots & \cdots & \cdots \\ \sigma_{k}^{2} & \sigma_{k}^{2} & \cdots & \ddots & \cdots & \cdots & \cdots & \ddots & \cdots \\ \cdots & \cdots & \cdots & \cdots & \cdots & \cdots & \cdots & \cdots & \ddots & \cdots \\ \sigma_{k}^{2} & \sigma_{k}^{2} & \cdots & \cdots & \sigma_{m}^{2} & \sigma_{k}^{2} & \cdots & \cdots & \cdots & \sigma_{m}^{2} \end{array} \end{array}\right|\\ & & =|COV(Y^{n},X^{-1})|. \end{array}$$

Consequently, $|COV\left(Y^{n},X^{-1}\right)|=|COV\left(Y^{n},X^{-2}\right)|$. Similarly, we can prove that $|COV\left(Y^{n},X^{-2}\right)|=|COV\left(Y^{n},X^{-3}\right)|=\ldots =|COV\left(Y^{n},X^{-n}\right)|$. That is to say, no matter what value i∈{1,2,…,n} takes, the result of the determinant $\left|COV(Y^{n},X^{-i})\right|$ is the same. So the proof of Lemma 1 is completed. □

Therefore, we could calculate $\left|COV(Y^{n},X^{-i})\right|$ as $\left|COV(Y^{n},X^{-1})\right|$,

(22) $$\begin{array}{ccc} & & |COV\left(Y^{n},X^{-i}\right)|=|COV\left(Y^{n},X^{-1}\right)|=\\ & & ={\left|\begin{array}{c} \begin{array}{cccccccc} \sigma_{m}^{2}+\sigma^{2} & \sigma_{k}^{2}+\sigma_{e}^{2} & \cdots & \sigma_{k}^{2}+\sigma_{e}^{2} & \sigma_{k}^{2} & \sigma_{k}^{2} & \cdots & \sigma_{k}^{2}\\ \sigma_{k}^{2}+\sigma_{e}^{2} & \sigma_{m}^{2}+\sigma^{2} & \cdots & \cdots & \sigma_{m}^{2} & \sigma_{k}^{2} & \cdots & \cdots \\ \cdots & \cdots & \ddots & \cdots & \cdots & \cdots & \ddots & \cdots \\ \sigma_{k}^{2}+\sigma_{e}^{2} & \cdots & \cdots & \sigma_{m}^{2}+\sigma^{2} & \sigma_{k}^{2} & \cdots & \cdots & \sigma_{m}^{2}\\ \sigma_{k}^{2} & \sigma_{m}^{2} & \cdots & \sigma_{k}^{2} & \sigma_{m}^{2} & \sigma_{k}^{2} & \cdots & \sigma_{k}^{2}\\ \sigma_{k}^{2} & \sigma_{k}^{2} & \cdots & \cdots & \sigma_{k}^{2} & \sigma_{m}^{2} & \cdots & \cdots \\ \cdots & \ddots & \cdots & \cdots & \cdots & \cdots & \ddots & \cdots \\ \sigma_{k}^{2} & \cdots & \cdots & \sigma_{m}^{2} & \sigma_{k}^{2} & \cdots & \cdots & \sigma_{m}^{2} \end{array} \end{array}\right|}_{\left(2n-1)\times (2n-1\right)}\\ & & =\left(\sigma_{m}^{2}+\sigma^{2}\right){\left(\sigma^{2}-\sigma_{e}^{2}\right)}^{n-1}{\left(\sigma_{m}^{2}-\sigma_{k}^{2}\right)}^{n-1}+{\left(-1\right)}^{n+1}\left(\sigma_{e}^{2}-\sigma_{m}^{2}-\sigma^{2}\right)\left(n-1\right)\sigma_{e}^{2}{\left(\sigma_{e}^{2}-\sigma^{2}\right)}^{n-2}{\left(\sigma_{m}^{2}-\sigma_{k}^{2}\right)}^{n-1}\\ & & +\sigma_{k}^{2}\left[\left(n-1\right)\sigma_{e}^{2}+\frac{\left(\sigma_{e}^{2}-\sigma^{2}\right)\sigma_{k}^{2}}{-\sigma_{k}^{2}-\sigma_{e}^{2}+\sigma_{m}^{2}+\sigma^{2}}\right]{\left(\sigma_{e}^{2}-\sigma^{2}\right)}^{n-2}\left[\left(n-1\right)\left(-\sigma_{k}^{2}-\sigma_{e}^{2}+\sigma_{m}^{2}+\sigma^{2}\right)\right]{\left(\sigma_{k}^{2}-\sigma_{m}^{2}\right)}^{n-2}. \end{array}$$

For $\left|COV(Z^{n})\right|$, we had

(23) $$\begin{array}{ccc} & & \left|COV(Z^{n})\right|\\ & & ={\left|\begin{array}{cccc} EZ_{1}^{2} & EZ_{1}Z_{2} & \cdots & EZ_{1}Z_{n}\\ EZ_{2}Z_{1} & EZ_{2}^{2} & \cdots & EZ_{2}Z_{n}\\ \vdots & \vdots & \cdots & \vdots \\ EZ_{n}Z_{1} & EZ_{n}Z_{2} & \cdots & EZ_{n}^{2} \end{array}\right|}_{n\times n}\\ & & ={\left|\begin{array}{cccc} \sigma^{2} & \sigma_{e}^{2} & \cdots & \sigma_{e}^{2}\\ \sigma_{e}^{2} & \sigma^{2} & \cdots & \sigma_{e}^{2}\\ \vdots & \vdots & \cdots & \vdots \\ \sigma_{e}^{2} & \sigma_{e}^{2} & \cdots & \sigma^{2} \end{array}\right|}_{n\times n}\\ & & =\left[\sigma^{2}+\left(n-1\right)\sigma_{e}^{2}\right]{\left(\sigma^{2}-\sigma_{e}^{2}\right)}^{n-1}. \end{array}$$

From (12), we calculated $|COV\left(X^{-i}\right)|=\left[\sigma_{m}^{2}+\left(n-2\right)\sigma_{k}^{2}\right]{\left(\sigma_{m}^{2}-\sigma_{k}^{2}\right)}^{n-2}$. Thus, (19) could be expressed as

(24) $$\begin{array}{ccc} & & I(X_{i};Y^{n}|X^{-i})\\ & & =\frac{1}{2}\log\left(\frac{A+B+C+D}{\left[\sigma_{m}^{2}+\left(n-2\right)\sigma_{k}^{2}\right]\left(\sigma^{2}+\left(n-1\right)\sigma_{e}^{2}\right)\left(\sigma^{2}-\sigma_{e}^{2}\right)}\right)\\ & & \leq \epsilon , \end{array}$$

where $A=\left(\sigma_{m}^{2}+\sigma^{2}\right)\left(\sigma^{2}-\sigma_{e}^{2}\right)\left(\sigma_{m}^{2}-\sigma_{k}^{2}\right)$, $B={\left(-1\right)}^{n+1}\left(n-1\right)\sigma_{e}^{2}\left(\sigma_{e}^{2}-\sigma_{m}^{2}-\sigma^{2}\right)\left(\sigma_{m}^{2}-\sigma_{k}^{2}\right)$, $C=\left(n-1\right)\sigma_{k}^{4}\left(\sigma_{e}^{2}-\sigma^{2}\right)$, and $D={\left(n-1\right)}^{2}\sigma_{e}^{2}\sigma_{k}^{2}\left(\sigma_{m}^{2}+\sigma^{2}-\sigma_{k}^{2}-\sigma_{e}^{2}\right)$

As shown, Equation (24) is very complicated, and it was difficult for us to directly derive the range of values of $\sigma^{2}$ and $\sigma_{e}^{2}$, the two parameters of the noise. Instead, we calculated the utility expression $U=\frac{I(X^{n};Y^{n})}{n}$, and then we used the nonlinear constraint optimization function to obtain the optimal values of $\sigma^{2}$ and $\sigma_{e}^{2}$ which could maximize the *U*.

Equation (4) could be further computed as

(25) $$\begin{array}{ccc} & & U=\frac{1}{n}I\left(X^{n};Y^{n}\right)\\ & & =\frac{1}{n}\left[h\left(Y^{n}\right)-h\left(Y^{n}|X^{n}\right)\right]\\ & & =\frac{1}{n}\left[h\left(Y^{n}\right)-h\left(Z^{n}\right)\right]\\ & & =\frac{1}{2n}{\log[\left(2\pi e\right)}^{n}|COV\left(Y_{1},\ldots ,Y_{n}\right)\left|\right]\\ & & -\frac{1}{2n}{\log[\left(2\pi e\right)}^{n}|COV\left(Z_{1},\ldots ,Z_{n}\right)\left|\right]\\ & & =\frac{1}{2n}\log\left(\frac{\left|COV(Y_{1},\ldots ,Y_{n})\right|}{\left|COV(Z_{1},\ldots ,Z_{n})\right|}\right). \end{array}$$

For $\left|COV(Y_{1},\ldots ,Y_{n})\right|$, we had

(26) $$\begin{array}{ccc} & & \left|COV(Y_{1},\ldots ,Y_{n})\right|\\ & & ={\left|\begin{array}{cccc} EY_{1}^{2} & EY_{1}Y_{2} & \cdots & EY_{1}Y_{n}\\ EY_{2}Y_{n} & EY_{2}^{2} & \cdots & EY_{2}Y_{n}\\ \vdots & \vdots & \cdots & \vdots \\ EY_{n}Y_{1} & EY_{n}Y_{2} & \cdots & EY_{n}^{2} \end{array}\right|}_{n\times n}\\ & & ={\left|\begin{array}{cccc} \sigma_{m}^{2}+\sigma^{2} & \sigma_{k}^{2}+\sigma_{e}^{2} & \cdots & \sigma_{k}^{2}+\sigma_{e}^{2}\\ \sigma_{k}^{2}+\sigma_{e}^{2} & \sigma_{m}^{2}+\sigma^{2} & \cdots & \sigma_{k}^{2}+\sigma_{e}^{2}\\ \vdots & \vdots & \cdots & \vdots \\ \sigma_{k}^{2}+\sigma_{e}^{2} & \sigma_{k}^{2}+\sigma_{e}^{2} & \cdots & \sigma_{m}^{2}+\sigma^{2} \end{array}\right|}_{n\times n}\\ & & =\left[\sigma_{m}^{2}+\sigma^{2}+\left(n-1\right)\left(\sigma_{k}^{2}+\sigma_{e}^{2}\right)\right]{\left(\sigma_{m}^{2}+\sigma^{2}-\sigma_{k}^{2}-\sigma_{e}^{2}\right)}^{n-1}. \end{array}$$

We calculated $|COV\left(Z^{n}\right)|=\left[\sigma^{2}+\left(n-1\right)\sigma_{e}^{2}\right]{\left(\sigma^{2}-\sigma_{e}^{2}\right)}^{n-1}$. Thus, (25) could be expressed as

(27) $$\begin{array}{ccc} & & U=\frac{1}{n}I\left(X^{n};Y^{n}\right)\\ & & =\frac{1}{2n}\log\left[\sigma_{m}^{2}+\sigma^{2}+\left(n-1\right)\left(\sigma_{k}^{2}+\sigma_{e}^{2}\right)\right]+\frac{1}{2n}\log\frac{{\left(\sigma_{m}^{2}+\sigma^{2}-\sigma_{k}^{2}-\sigma_{e}^{2}\right)}^{n-1}}{\left[\sigma^{2}+\left(n-1\right)\sigma_{e}^{2}\right]{\left(\sigma^{2}-\sigma_{e}^{2}\right)}^{n-1}}. \end{array}$$

The next step was to combine (24) with (27). The aim was to find the most suitable values of $\sigma^{2}$ and $\sigma_{e}^{2}$ so that (27) could obtain the maximum value under the condition of (24). In order to solve this problem, we used MATLAB’s nonlinear optimization function for the simulation. We varied the privacy budget ϵ values and searched for $\sigma^{2}$ and $\sigma_{e}^{2}$ that would maximize utility.

**Figure 6:**
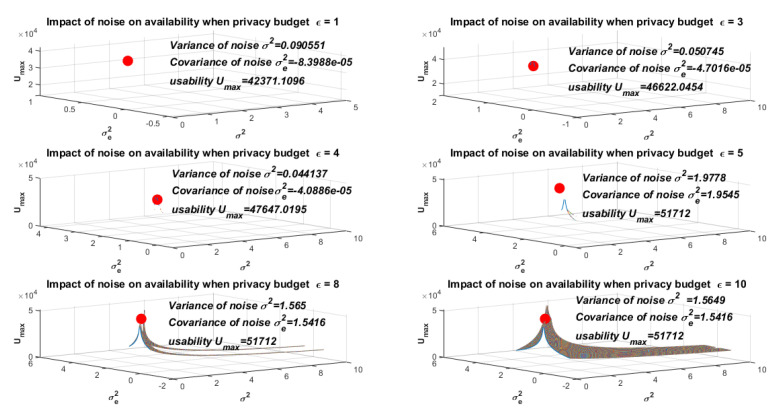
The impact of noise level on utility with different privacy budget ϵ.

**Figure 7:**
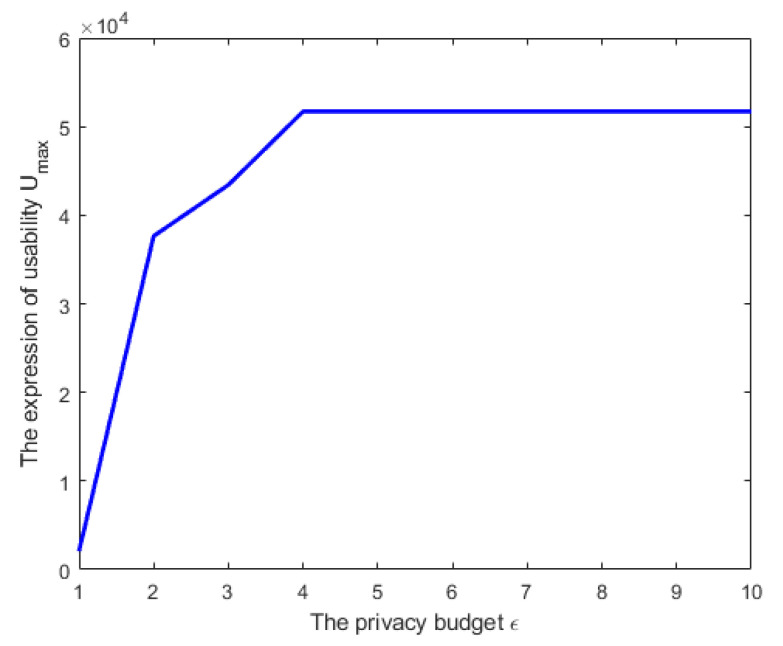
The relationship between utility and privacy in Case 3.

Figure 6 shows the simulation results when the privacy budget ϵ takes different values (ϵ={1,3,4,5,8,10}). In this figure, we assumed that $\sigma_{m}^{2}=30$, $\sigma_{k}^{2}=2$, and n=101. We found that when the privacy budget ϵ increased within a certain range, the value for measuring utility also increased. However, there was an upper bound, that is, when the privacy budget ϵ was out of range, the value of $U_{max}$ remained unchanged. Figure 7 plots the relationship between $U_{max}$ and ϵ. In Figure 7, we obtained the value of the expression of utility when the privacy budget ϵ={1,2,3,4,5,6,7,8,9,10}. In this figure, we assumed that $\sigma_{m}^{2}=30$, $\sigma_{k}^{2}=2$, and n=101. The ten points (ϵ={1,2,3,4,5,6,7,8,9,10}) were connected into a line, and finally we obtained the relationship trend between $U_{max}$ and ϵ.

## 4. Conclusions

In this paper, we used the DP mechanism to protect the clients’ local parameters. From an information-theoretic point of view, we studied the utility–privacy trade-off in DML with the help of the DP mechanism. Specifically, three cases including independent clients’ local parameters with independent DP noise and dependent clients’ local parameters with independent/dependent DP noise were considered. Mutual information and conditional mutual information were used to characterize utility and privacy, respectively. First, we showed the relationship between utility and privacy for the three cases. Then, we show the optimal noise variance that achieved the maximal utility under a certain level of privacy. Finally, the results of this paper were further illustrated by numerical results.

The limitations of this paper are that the local parameters and the added noise of the client were only assumed to be Gaussian distributed, and multiround model training was not considered, which we will in our future work. 

## Data Availability

The data used in this work are available from the corresponding author upon reasonable request.
